# Supplementary material for: Temporal trends of hospitalizations, comorbidity burden and in-hospital outcomes in patients admitted with asthma in the United States: Population-based study
Source: PLoS One. 2022 Dec 14;17(12):e0276731. doi: 10.1371/journal.pone.0276731 (PMC9750011; doi:10.1371/journal.pone.0276731)
Supplement: S1 Table — (PDF) [file pone.0276731.s001.pdf]

Supplementary data to "Temporal trends of hospitalizations, comorbidity burden and in-hospital outcomes in patients admitted with asthma in the US" Zghebi SS, Mamas MA, Mohamed MO, Kontopantelis E. 2022

S1 Table. Annual trends of admissions and baseline characteristics of patients admitted with asthma between 2004 and 2017

	Overall (2004-17)	2004	2005	2006	2007	2008	2009	2010	2011	2012	2013	2014	2015	2016	2017
Weighted admissions (N)	3,098,863	260,003	270,750	248,919	224,714	214,789	245,772	223,147	200,524	233,520	212,685	207,800	189,735	185,565	180,940
Age categories, %															
• <5 years	23.71	28.22	27.01	25.85	24.96	23.58	24.09	24.84	22.21	23.47	22.43	22.07	20.95	19.40	18.76
• 5-17 years	22.76	20.78	23.51	21.87	21.31	20.31	22.96	21.95	20.93	24.66	24.11	26.67	24.08	23.11	22.81
• 18-39 years	17.70	17.93	16.37	17.87	18.32	18.79	18.27	17.61	18.46	17.48	17.14	16.93	17.62	17.66	17.57
• 40-59 years	22.18	21.51	21.10	22.58	23.45	23.67	22.46	22.85	23.51	21.12	21.56	20.49	21.81	22.57	22.20
• 60-79 years	10.19	8.56	8.63	8.73	8.87	9.82	9.18	9.48	11.02	9.93	10.96	10.52	11.80	13.36	14.35
• ≥80 years	3.46	3.00	3.37	3.10	3.08	3.82	3.03	3.27	3.87	3.34	3.79	3.32	3.74	3.90	4.31
Smokers, %	11.97	9.55	10.7	11.4	12.7	13.69	13.6	14.9	15.7	14.9	15.62	16.47	13.86	1.12	0.96
In-hospital deaths, %	0.11	0.10	0.08	0.09	0.11	0.08	0.09	0.11	0.11	0.10	0.12	0.17	0.14	0.17	0.18
Location of patient's residence, %															
• Central counties	12.7	NA	NA	NA	NA	NA	NA	NA	NA	NA	39.34	40.52	39.98	40.28	40.68
• Fringe counties	7.5	NA	NA	NA	NA	NA	NA	NA	NA	NA	23.78	23.69	23.27	23.76	24.07
• Counties <1m popltn	8.0	NA	NA	NA	NA	NA	NA	NA	NA	NA	24.87	25.01	25.88	25.88	25.01
• Micropolitan counties	2.0	NA	NA	NA	NA	NA	NA	NA	NA	NA	7.41	6.14	6.24	5.89	5.89
• Not metropolitan/ micropolitan counties	1.3	NA	NA	NA	NA	NA	NA	NA	NA	NA	4.39	4.41	4.40	4.00	4.07
• Unknown	68.6	NA	NA	NA	NA	NA	NA	NA	NA	NA	0.21	0.23	0.23	0.19	0.28
Intubation/ventilator, %	4.27	3.66	3.04	3.52	2.9	3.25	3.1	3.74	3.7	4.4	5.02	6.10	6.32	6.16	6.71
Nebuliser treatment, %	8.14	11.1	7.6	11.1	9.2	6.49	11.4	6.72	5.5	7.5	7.51	7.64	7.78	6.49	5.92
Tracheostomy, %	0.04	0.02	0.04	0.04	0.04	0.04	0.03	0.04	0.04	0.03	0.05	0.05	0.07	0.06	0.09
Chest CT scan, %	0.47	0.42	0.44	0.42	0.7	0.65	0.52	0.55	0.62	0.52	0.50	0.51	0.41	0.15	0.11
Any respiratory procedure, %	12.43	13.9	10.2	13.92	12.4	10.0	14.5	10.91	10.05	12.0	12.71	13.69	14.10	12.86	12.83
Admissions month, %															
• January	9.3	9.2	9.4	9.2	9.6	9.6	8.9	9.7	9.6	8.9	9.9	8.6	9.7	9.0	9.6

	Overall (2004-17)	2004	2005	2006	2007	2008	2009	2010	2011	2012	2013	2014	2015	2016	2017
• February	8.9	9.5	9.4	9.0	8.7	10.5	8.5	9.1	9.3	8.8	8.6	7.3	8.4	9.2	8.9
• March	9.5	9.7	9.5	9.7	9.4	9.2	8.6	10.0	10.4	9.4	9.4	8.6	9.5	9.8	9.5
• April	8.7	8.5	8.7	7.9	8.5	8.9	8.0	8.8	9.1	9.1	9.1	8.4	9.2	8.9	9.3
• May	8.7	8.6	8.9	9.0	8.8	8.2	7.9	8.6	9.1	7.8	9.5	8.1	9.0	8.8	9.4
• June	5.7	5.8	5.6	5.4	5.6	5.5	6.0	5.6	5.6	5.2	6.0	5.6	5.9	6.1	5.7
• July	4.7	4.7	4.5	4.6	4.9	4.7	4.5	4.7	4.4	4.6	4.8	5.1	4.8	5.4	4.8
• August	6.4	6.2	5.9	5.7	6.5	6.1	6.7	6.0	5.6	7.0	6.1	8.6	6.3	7.1	6.2
• September	9.7	8.4	8.5	10.3	9.4	10.0	12.1	9.8	8.8	10.3	9.0	12.8	8.8	8.8	9.1
• October	9.8	9.8	11.2	10.3	9.9	9.3	11.1	10.0	9.5	9.9	9.2	9.0	9.5	8.9	9.0
• November	9.4	9.9	9.9	9.6	10.1	8.7	9.4	9.2	9.5	9.2	9.2	8.5	9.4	9.0	9.2
• December	9.1	9.8	8.6	9.3	8.6	9.2	8.5	8.5	9.2	9.9	9.0	9.4	9.4	9.0	9.4
Hospital bed size (indicator of hospital size), %															
• Small	15.6	12.6	15.9	16.2	16.4	13.4	14.7	11.1	11.5	15.6	14.4	19.1	19.3	19.6	21.6
• Medium	27.8	27.4	29.8	26.6	25.3	25.5	27.0	25.9	25.5	28.9	29.1	29.9	29.6	29.5	29.6
• Large	56.3	60.0	54.3	57.1	58.2	60.9	57.0	62.0	61.5	55.6	56.5	51.0	51.2	50.8	48.7
• Unknown	0.3	0.00	0.00	0.2	0.1	0.2	1.39	1.1	1.52	0.00	0.00	0.00	0.00	0.00	0.00
Control /ownership of hospital, %															
• Government/non-federal	8.89	NA	NA	NA	NA	11.51	17.29	13.57	11.25	13.06	12.81	12.75	13.42	12.86	12.18
• Private/ non-profit	49.75	NA	NA	NA	NA	77.94	65.08	76.11	74.81	73.54	73.77	74.30	73.33	73.63	75.09
• Private/investor-own	8.64	NA	NA	NA	NA	10.39	16.24	9.23	12.43	13.40	13.43	12.95	13.24	13.52	12.74
• Unknown	32.71	NA	NA	NA	NA	0.16	1.39	1.09	1.52	0.00	0.00	0.00	0.00	0.00	0.00
Hospital location/ teaching status, %															
• Rural	11.5	15.2	14.0	14.2	15.0	13.3	12.5	10.6	13.2	10.3	9.0	7.6	7.8	7.0	6.9
• Urban/Non-teaching	30.8	37.5	37.3	33.5	35.2	36.9	32.3	33.3	34.2	30.7	30.6	20.3	21.7	20.9	17.9
• Urban/Teaching	57.4	47.3	48.8	52.2	49.6	49.3	53.8	55.0	51.1	59.1	60.4	72.0	70.5	72.1	75.2
• Unknown	0.3	0.00	0.00	0.2	0.1	0.2	1.4	1.1	1.5	0.00	0.00	0.00	0.00	0.00	0.00
