# Supplementary material for: Temporal trends of hospitalizations, comorbidity burden and in-hospital outcomes in patients admitted with asthma in the United States: Population-based study
Source: PLoS One. 2022 Dec 14;17(12):e0276731. doi: 10.1371/journal.pone.0276731 (PMC9750011; doi:10.1371/journal.pone.0276731)
Supplement: S2 Table — (PDF) [file pone.0276731.s002.pdf]

S2 Table. Comparison of asthma admissions before and after the ICD code switch by calendar quarters in 2015

	ICD-9 coding use				ICD-10 coding use
	Jan - September 2015 (Calendar quarters 1-3)				October 2015 (Calendar quarter 4)
	Q1	Q2	Q3	Full Q 1-3	
Weighted admissions (N)	52,500	46,690	36,995	136,185	53,550
Admissions per 100,000 population	65.3	58.1	46.0	56.5	66.6
