# Supplementary material for: Temporal trends of hospitalizations, comorbidity burden and in-hospital outcomes in patients admitted with asthma in the United States: Population-based study
Source: PLoS One. 2022 Dec 14;17(12):e0276731. doi: 10.1371/journal.pone.0276731 (PMC9750011; doi:10.1371/journal.pone.0276731)
Supplement: S3 Table — (PDF) [file pone.0276731.s003.pdf]

S3 Table. Comparison of main patients' characteristics before and after the ICD code switch in 2015

	**ICD-9 coding use (Jan 2004 – Sept 2015)**	**ICD-10 coding use (Oct 2015 – Dec 2017)**
Age, mean (±SD)	28.12 (±25)	31.29(±26)
Female, %	56.68	58.62
Ethnicity, %<br>• White	35.63	36.86
• Black *	26.41	32.91
• Hispanic	15.26	18.13
• Asian/Pacific Islander	1.89	2.69
• Native American	0.67	0.73
• Other	3.87	4.51
• Unknown**	16.27	4.16
Asthma severity, %<br>• Minor	52.92	42.62
• Moderate	37.65	37.11
• Major***	8.23	17.87
• Extreme	1.19	2.39

*The annual proportion of Black patients was already increasing before the code switch (increased from 23% in 2004 to 32.4% in 2014 i.e. in the ICD-9 phase) (Table 1)

**The annual proportion of patients with unknown ethnicity was already declining before the code switch (declined from 26% in 2004 to 5% in 2014 i.e. in the ICD-9 phase) (Table 1)

***The annual proportion of patients with major severity was already increasing before the code switch (increased from 6.4% in 2004 to 11.1% in 2014 i.e. in the ICD-9 phase) (Table 1)

We assessed if the patients selected before and after the ICD code switch differed, but we found no differences that can be attributed to that switch as shown in the Table above.
