# Supplementary material for: Temporal trends of hospitalizations, comorbidity burden and in-hospital outcomes in patients admitted with asthma in the United States: Population-based study
Source: PLoS One. 2022 Dec 14;17(12):e0276731. doi: 10.1371/journal.pone.0276731 (PMC9750011; doi:10.1371/journal.pone.0276731)
Supplement: S4 Table — a Cardiovascular disease: any of myocardial infarction, coronary heart disease, heart valve disease, peripheral vascular disease, heart failure, atrial fibrillation/flutter, TIA/stroke. RA: rheumatoid arthritis; COPD: chronic obstructive pulmonary disease; GERD: gastroesophageal reflux disease; TIA: transient ischaemic attack. (PDF) [file pone.0276731.s004.pdf]

Supplementary data to "Temporal trends of hospitalizations, comorbidity burden and in-hospital outcomes in patients admitted with asthma in the US"
Zghebi SS, Mamas MA, Mohamed MO, Kontopantelis E. 2022

S4 Table. Annual comorbidity profile in patients admitted with asthma between 2004 and 2017

	Weighted proportion (%)	Overall	2004	2005	2006	2007	2008	2009	2010	2011	2012	2013	2014	2015	2016	2017	*P*-value
1.	Hypertension	23.18	17.6	18.5	20.0	21.8	23.4	22.5	23.6	25.8	23.5	24.6	23.9	26.5	28.8	29.6	0.000
2.	Obesity	12.86	7.6	8.5	8.8	9.7	10.9	11.4	12.4	14.2	14.1	15.4	15.8	17.6	19.1	20.3	0.000
3.	Diabetes	12.74	9.8	10.6	11.1	11.9	12.6	13.2	13.4	14.6	12.7	13.4	12.7	13.9	15.1	15.8	0.000
4.	GERD	12.01	9.3	10.0	10.1	10.7	11.9	11.0	12.0	13.6	11.9	12.7	12.2	14.4	15.8	15.7	0.000
5.	Dyslipidaemia	9.63	5.2	6.5	7.2	8.4	9.2	9.2	10.0	11.5	10.3	11.3	10.7	12.0	13.1	14.1	0.000
6.	Cardiovascular disease [a]	8.44	6.6	7.0	7.2	7.4	8.5	7.6	8.2	9.4	8.1	9.2	8.5	10.0	11.1	12.0	0.000
7.	Depression	6.77	5.4	5.6	5.9	6.3	6.7	6.4	7.1	8.0	0.8	6.9	7.1	7.5	8.3	8.5	0.000
8.	Hypothyroidism	4.89	3.5	3.8	3.8	4.3	4.8	4.6	4.8	5.5	5.0	5.5	5.5	6.0	6.3	6.7	0.000
9.	Obstructive sleep apnoea	4.74	0.00	0.5	2.6	3.5	4.1	4.4	5.4	6.1	5.9	6.6	6.5	7.5	8.5	9.0	0.000
10.	Anemia	4.70	3.3	3.4	3.7	4.2	4.7	4.6	4.9	5.4	5.2	5.4	5.0	5.3	6.1	6.0	0.000
11.	Coronary heart disease	3.64	2.9	3.3	3.5	3.6	3.8	3.4	3.5	4.1	3.6	3.9	3.5	3.8	4.4	4.5	0.000
12.	Heart failure	3.40	3.0	3.0	3.1	3.0	3.2	2.8	3.2	3.5	2.9	3.4	3.3	4.1	4.7	5.3	0.000
13.	Psychoses	2.93	2.0	2.0	2.3	2.6	2.9	3.0	3.4	3.5	3.2	3.2	3.3	3.6	3.3	3.5	0.000
14.	COPD	2.75	1.8	2.1	2.2	1.9	2.2	2.1	2.1	2.3	2.1	2.1	2.2	3.4	7.3	6.8	0.000
15.	Chronic sinusitis	2.66	2.9	3.2	2.9	2.9	3.2	2.7	2.9	3.2	2.5	2.4	2.2	2.2	1.9	1.8	0.000
16.	Atrial fibrillation/flutter	1.91	1.3	1.5	1.5	1.6	1.9	1.7	1.8	2.3	2.1	2.4	2.2	2.5	2.1	2.1	0.000
17.	Chronic kidney disease	1.88	0.2	0.3	0.8	1.4	1.8	1.7	1.9	2.5	2.2	2.7	2.5	2.9	3.4	3.7	0.000
18.	RA/collagen vascular diseases	1.34	0.9	1.0	0.9	1.1	1.2	1.3	1.26	1.5	1.4	1.5	1.5	1.7	2.0	2.0	0.000
19.	Heart valve disease	0.99	0.7	0.8	0.8	0.9	0.9	0.8	0.8	1.0	0.9	0.9	1.0	1.3	1.6	1.8	0.000
20.	Myocardial infarction	0.96	0.7	0.8	0.8	0.8	1.0	0.9	0.9	1.1	1.0	1.0	0.9	1.1	1.5	1.4	0.000
21.	TIA/Stroke	0.93	0.00	0.00	0.00	0.1	1.0	0.9	1.2	1.3	1.3	1.4	1.4	1.6	1.9	2.0	0.000
22.	Liver disease	0.76	0.5	0.6	0.6	0.6	0.7	0.7	0.8	0.8	0.8	0.8	0.8	1.01	1.1	1.2	0.000
23.	Coagulopathy	0.63	0.4	0.4	0.4	0.4	0.6	0.5	0.6	0.7	0.8	0.9	0.8	0.9	0.9	1.0	0.000
24.	Atopic dermatitis	0.61	0.5	0.5	0.5	0.5	0.4	0.5	0.6	0.5	0.7	0.8	0.9	0.8	0.8	1.0	0.000
25.	Cancer	0.55	0.5	0.4	0.4	0.5	0.6	0.5	0.6	0.6	0.5	0.6	0.6	0.7	0.7	0.7	0.000
26.	Peripheral vascular disease	0.54	0.3	0.4	0.4	0.5	0.5	0.4	0.5	0.6	0.5	0.7	0.6	0.7	0.7	0.9	0.000
27.	Weight loss	0.37	0.2	0.2	0.2	0.2	0.3	0.3	0.3	0.5	0.4	0.4	0.4	0.5	0.7	0.8	0.000
28.	Lung cancer	0.10	0.1	0.1	0.1	0.1	0.1	0.1	0.1	0.1	0.1	0.1	0.1	0.1	0.1	0.1	0.000

[a] Cardiovascular disease: any of myocardial infarction, coronary heart disease, heart valve disease, peripheral vascular disease, heart failure, atrial fibrillation/flutter, TIA/stroke. RA: rheumatoid arthritis; COPD: chronic obstructive pulmonary disease; GERD: gastroesophageal reflux disease; TIA: transient ischaemic attack.
