# Supplementary material for: Temporal trends of hospitalizations, comorbidity burden and in-hospital outcomes in patients admitted with asthma in the United States: Population-based study
Source: PLoS One. 2022 Dec 14;17(12):e0276731. doi: 10.1371/journal.pone.0276731 (PMC9750011; doi:10.1371/journal.pone.0276731)
Supplement: S5 Table — a Comorbidities identified as significant predictors for in-hospital intubation/mechanical ventilation from univariable regression models per outcome. RA: rheumatoid arthritis; COPD: chronic obstructive pulmonary disease; CVD: cardiovascular disease. (PDF) [file pone.0276731.s005.pdf]

**S5 Table. Odds ratio (95% CI) for in-hospital intubation or mechanical ventilation in adult patients (18+ years) admitted with asthma in 2004, 2010 and 2017**

| | 2004 | 2010 | 2017 |
|---|---|---|---|
| Age | 0.99 (0.98; 0.99) | 0.98 (0.98; 0.99) | 0.98 (0.98; 0.99) |
| Male | Ref | Ref | Ref |
| Female | 0.81 (0.68; 0.95) | 0.86 (0.75; 0.99) | 0.89 (0.80; 1.00) |
| Race | | | |
| White | Ref | Ref | Ref |
| Black | 1.18 (0.97; 1.43) | 1.86 (1.60; 2.15) | 2.01 (1.77; 2.28) |
| Hispanic | 1.15 (0.89; 1.48) | 1.81 (1.48; 2.22) | 1.55 (1.32; 1.83) |
| Asian/Pacific Islander | 1.78 (1.09; 2.93) | 2.52 (1.66; 3.82) | 2.35 (1.73; 3.20) |
| Native American | 0.87 (0.30; 2.53) | 0.73 (0.29; 1.83) | 1.71 (0.99; 2.96) |
| Other | 0.80 (0.46; 1.42) | 1.39 (0.93; 2.09) | 1.74 (1.33; 2.28) |
| Unknown | 0.83 (0.68; 1.02) | 0.63 (0.48; 0.84) | 0.90 (0.63; 1.30) |
| Weekend admission | 1.10 (0.93; 1.30) | 1.03 (0.90; 1.20) | 0.90 (0.79; 1.01) |
| Admission quarter | | | |
| Jan – Mar | Ref | Ref | Ref |
| Apr – Jun | 0.99 (0.80; 1.21) | 1.11 (0.93; 1.33) | 1.03 (0.89; 1.19) |
| Jul – Sep | 1.20 (0.96; 1.48) | 1.16 (0.96; 1.39) | 1.16 (1.00; 1.35) |
| Oct – Dec | 0.98 (0.80; 1.21) | 1.09 (0.91; 1.30) | 1.06 (0.92; 1.22) |
| Elective admission | 0.37 (0.24; 0.57) | 0.67 (0.47; 0.96) | 0.64 (0.44; 0.94) |
| Asthma severity (degree of loss of function) | | | |
| Minor | Ref | Ref | Ref |
| Moderate | 1.80 (1.48; 2.18) | 1.63 (1.37; 1.94) | 1.42 (1.19; 1.69) |
| Major | 8.64 (6.29; 10.9) | 3.64 (2.95; 4.48) | 4.33 (3.64; 5.14) |
| Extreme | 467.0 (342.2; 637.2) | 77.7 (61.3; 98.6) | 60.7 (48.9; 75.2) |
| Comorbidities [a] | | | |
| Diabetes | - | 0.94 (0.81; 1.10) | - |
| Hypothyroidism | 0.60 (0.41; 0.89) | - | 0.71 (0.58; 0.87) |
| Anemias | 1.07 (0.78; 1.47) | 0.92 (0.73; 1.15) | 0.97 (0.81; 1.16) |
| RA/collagen vascular disease | 0.44 (0.23; 0.84) | - | - |
| Weight loss | 0.34 (0.11; 1.07) | 0.59 (0.30; 1.15) | - |
| Obesity | 1.27 (1.04; 1.56) | 0.84 (0.72; 0.99) | 0.86 (0.76; 0.98) |
| COPD | - | - | 1.13 (0.95; 1.33) |
| Obstructive sleep apnoea | - | 3.14 (2.62; 3.77) | 2.19 (1.89; 2.54) |
| Dyslipidaemia | - | - | 1.00 (0.86; 1.16) |
| Coagulopathies | 0.43 (0.21; 0.88) | - | 0.77 (0.53; 1.11) |
| Hypertension | - | - | 0.95 (0.83; 1.08) |
| CVD | 0.65 (0.50; 0.85) | - | - |

[a] Comorbidities identified as significant predictors for in-hospital intubation/mechanical ventilation from univariable regression models per year. RA: rheumatoid arthritis; COPD: chronic obstructive pulmonary disease; CVD: cardiovascular disease
