# Supplementary material for: Temporal trends of hospitalizations, comorbidity burden and in-hospital outcomes in patients admitted with asthma in the United States: Population-based study
Source: PLoS One. 2022 Dec 14;17(12):e0276731. doi: 10.1371/journal.pone.0276731 (PMC9750011; doi:10.1371/journal.pone.0276731)
Supplement: S6 Table — (PDF) [file pone.0276731.s006.pdf]

S6 Table. Predictors of inflation-adjusted total hospitalization charges for adult patients (18+ years) admitted with asthma in 2004, 2010 and 2017

	2004	2010	2017
Age	68.6 (53.5; 83.8)	22.8 (-1.19; 46.7)	64.4 (32.6; 96.3)
Male<br>Female	Ref<br>953.0 (461.7; 1444.4)	Ref<br>1091.3 (348.8; 1833.8)	Ref<br>2192.2 (1005.0; 3379.5)
Race			
White	Ref	Ref	Ref
Black	-383.3 (-1009.4; 242.9)	-88.7 (-1082.9; 305.6)	1403.0 (419.7; 2386.3)
Hispanic	5231.1 (4345.5; 6116.8)	4856.5 (3880.0; 5832.9)	7975.0 (6224.6; 9725.4)
Asian/Pacific Islander	3574.6 (1978.4; 5170.8)	6710.3 (4183.5; 9237.2)	10346.0 (6761.9; 13930.0)
Native American	-1959.3 (-4241.8; 323.2)	190.0 (-2617.9; 2997.8)	1416.4 (-4882.4; 2049.5)
Other	365.0 (-1071.4; 1801.5)	1897.9 (-2146.4; 5942.3)	6483.6 (4034.8; 8932.3)
Unknown	-22291.3 (-2806.5; -1776.1)	-3141.3 (-3923.3; -2359.4)	-5178.9 (-7343.0; -3014.9)
Weekend admission	-290.9 (-794.7; 212.8)	21.7 (-677.6; 721.0)	143.2 (-1081.7; 1368.1)
Admission quarter			
Jan – Mar	Ref	Ref	Ref
Apr – Jun	-734.0 (-1415.6; -52.5)	-621.9 (-1437.4; 193.5)	-318.6 (-1548.2; 910.9)
Jul – Sep	-781.3 (-1408.6; -154.1)	-403.2 (-1313.2; 506.8)	-682.1 (-1929.1; 564.8)
Oct – Dec	-245.0 (-815.0; 325.2)	-457.8 (-1286.5; 370.9)	584.3 (-630.5; 1799.2)
Elective admission	-1390.0 (-2055.2; -724.8)	-3402.6 (-4286.3; -2519.0)	-2789.5 (-4607.0; -972.0)
Asthma severity (degree of loss of function)			
Minor	Ref	Ref	Ref
Moderate	3750.6 (3294.5; 4206.8)	3312.3 (2715.6; 3908.9)	3070.9 (2174.4; 3967.4)
Major	11394.8 (10008.2; 12781.4)	11680.3 (10339.9; 13020.7)	10651.1 (9377.8; 11924.5)
Extreme	42129.8 (34322.5; 49937.1)	45634.6 (37895.7; 53373.5)	57734.0 (50480.0; 64988.0)
Comorbidities [a]			
Diabetes	-1036.1 (-1705.5; -366.7)	5.49 (-844.7; 855.7)	546.8 (-638.7; 1732.4)
Hypothyroidism	-30.6 (-876.9; 815.7)	86.6 (-935.2; 1108.3)	1689.1 (-70.7; 3448.8)
Anemias	2458.0 (1214.7; 3701.3)	4850.9 (3490.6; 6211.1)	3203.2 (1284.5; 5121.9)
RA/collagen vascular disease	604.5 (-1554.4; -2763.4)	954.6 (-804.5; 2713.7)	-2838.9 (375.2; 5302.5)
Liver disease	-1612.9 (-3364.1; 138.3)	2530.9 (-1204.3; 6266.1)	1420.5 (-2008.8; 4849.8)
CKD	3618.9 (-1541.1; 8778.9)	-654.7 (-2947.2; 1637.7)	774.1 (-2072.4; 3620.6)
Psychoses	1281.4 (148.8; 2414.1)	1978.4 (674.9; 3282.0)	-
Depression	-	2.66 (-753.3; 758.6)	-
Weight loss	10151.1 (-2746.3; 23048.5)	4822.9 (-475.4; 10121.2)	13920.7 (2116.0; 25725.3)
Obesity	404.3 (-225.3; 1033.9)	714.8 (-8.66; 1438.2)	1638.1 (615.7; 2660.5)
Chronic sinusitis	2638.3 (1722.2; 3554.3)	3407.2 (2141.3; 4673.1)	8307.7 (4362.6; 12252.8)
COPD	-	-	3143.7 (1377.5; 4910.0)
Obstructive sleep apnoea	-	2336.8 (1201.6; 3471.9)	2757.5 (1079.3; 4435.8)
GERD	177.9 (-320.2; 676.0)	841.1 (153.9; 1528.3)	-443.7 (-1542.8; 655.5)
Cancer	868.7 (-1681.3; 3418.7)	1130.3 (-1366.4; 3627.0)	-
Dyslipidaemia	-328.9 (-954.7; 297.1)	943.8 (122.4; 1765.2)	-139.1 (-1306.9; 1028.7)
Coagulopathies	4014.6 (-188.2; 8217.5)	11201.4 (-398.2; 22801.0)	10731.6 (2059.6; 19403.6)
Hypertension	994.8 (455.1; 1534.4)	1044.7 (414.2; 1675.1)	1405.2 (283.1; 2527.4)
CVD	477.7 (-457.4; 1412.7)	1409.8 (34.4; 2785.1)	2649.0 (1260.0; 4038.0)

[a] comorbidities identified as significant predictors of costs from univariable regression models per year. RA: rheumatoid arthritis; CKD: chronic kidney disease; COPD: chronic obstructive pulmonary disease; GERD: gastroesophageal reflux disease; CVD: cardiovascular disease
