# Supplementary material for: Temporal trends of hospitalizations, comorbidity burden and in-hospital outcomes in patients admitted with asthma in the United States: Population-based study
Source: PLoS One. 2022 Dec 14;17(12):e0276731. doi: 10.1371/journal.pone.0276731 (PMC9750011; doi:10.1371/journal.pone.0276731)

Supplementary data to "Temporal trends of hospitalizations, comorbidity burden and in-hospital outcomes in patients admitted with asthma in the US" Zghebi SS, Mamas MA, Mohamed MO, Kontopantelis E. 2022

S1 Fig. Trends of length of stay (LOS) (panels A, B) and total costs (C, D) by sex and asthma severity of people admitted with asthma between 2004 and 2017

(A)

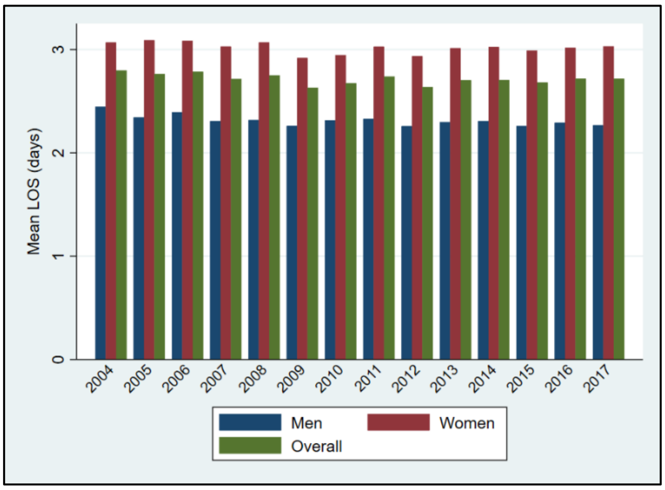

(B)

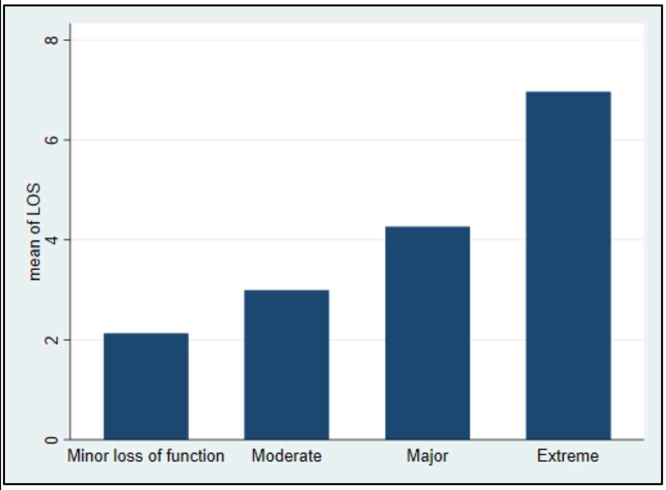

(C)

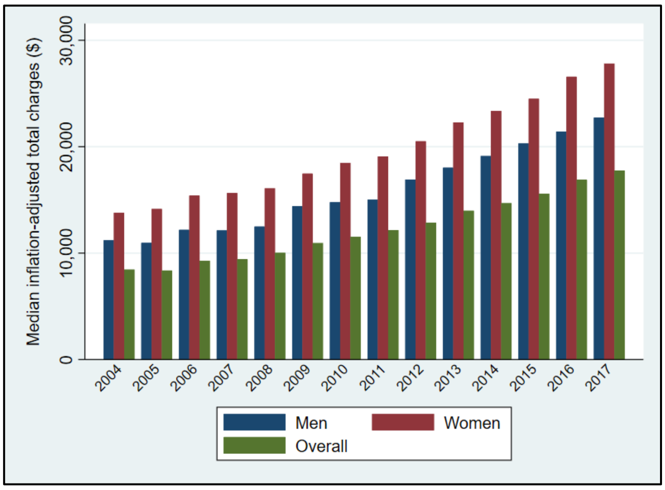

(D)

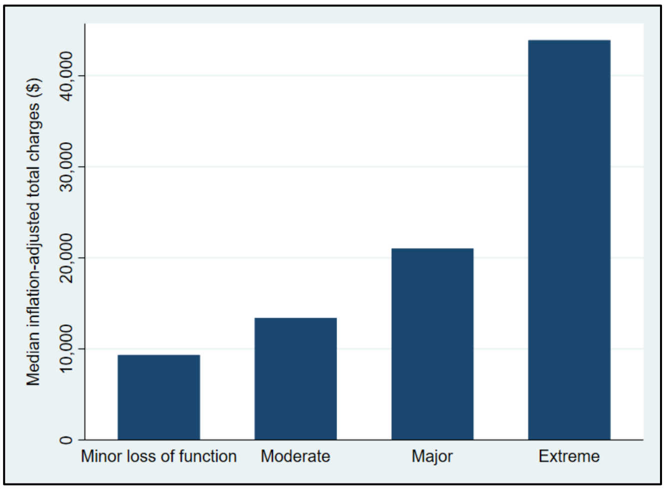

Supplement: S1 Fig — Trends of length of stay (LOS) (panels A, B) and total costs (C, D) by sex and asthma severity of people admitted with asthma between 2004 and 2017. (PDF) [file pone.0276731.s007.pdf]
