# Supplementary material for: Temporal trends of hospitalizations, comorbidity burden and in-hospital outcomes in patients admitted with asthma in the United States: Population-based study
Source: PLoS One. 2022 Dec 14;17(12):e0276731. doi: 10.1371/journal.pone.0276731 (PMC9750011; doi:10.1371/journal.pone.0276731)

Supplementary data to "Temporal trends of hospitalizations, comorbidity burden and in-hospital outcomes in patients admitted with asthma in the US" Zghebi SS, Mamas MA, Mohamed MO, Kontopantelis E. 2022

S2 Fig. Trends of in-hospital mortality by age of death, gender, asthma severity, and race in patients admitted between 2004 and 2017

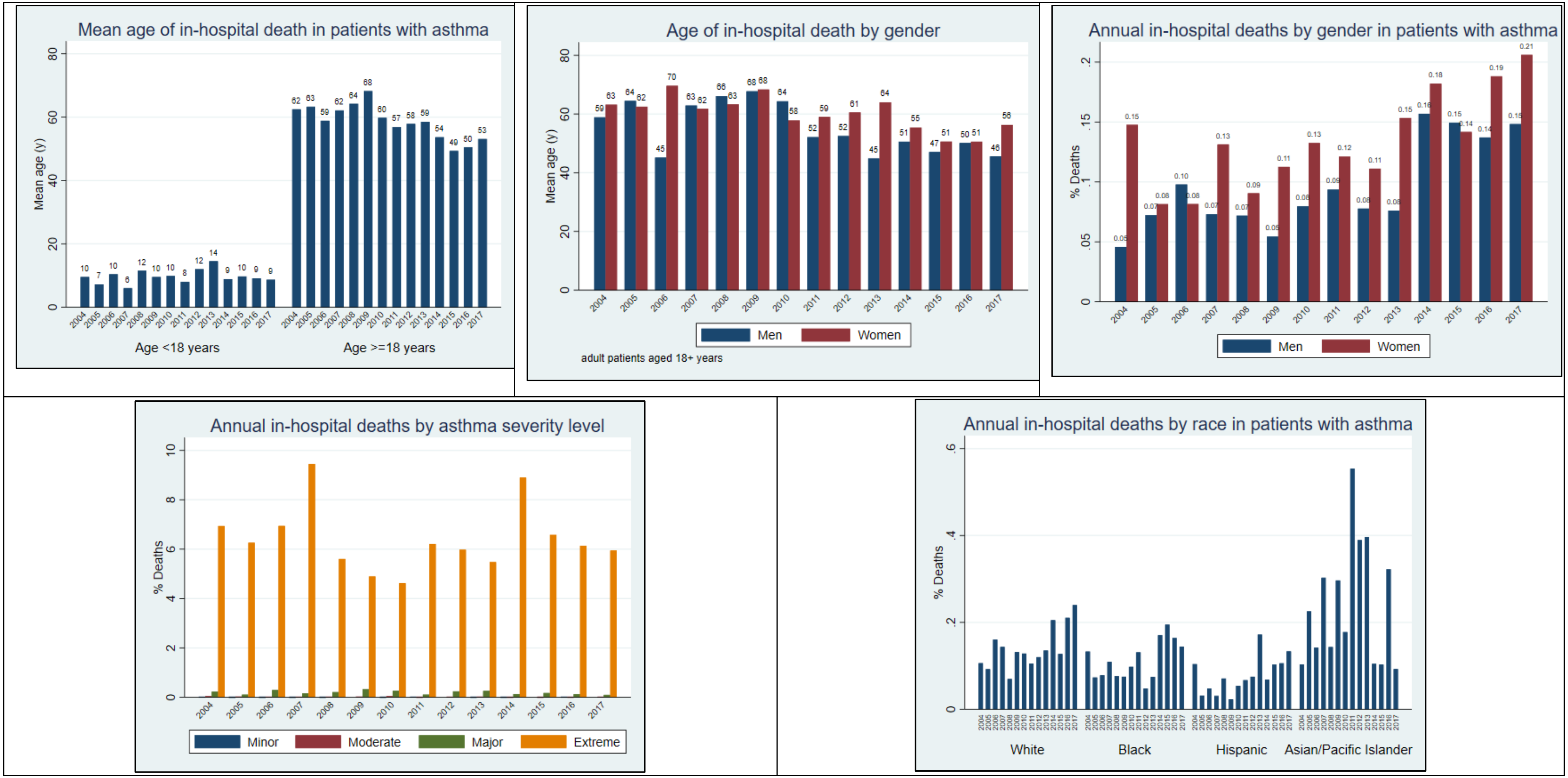

Supplement: S2 Fig — (PDF) [file pone.0276731.s008.pdf]
