# Supplementary material for: Temporal trends of hospitalizations, comorbidity burden and in-hospital outcomes in patients admitted with asthma in the United States: Population-based study
Source: PLoS One. 2022 Dec 14;17(12):e0276731. doi: 10.1371/journal.pone.0276731 (PMC9750011; doi:10.1371/journal.pone.0276731)
Supplement: S3 Fig — (PDF) [file pone.0276731.s009.pdf]

Supplementary data to "Temporal trends of hospitalizations, comorbidity burden and in-hospital outcomes in patients admitted with asthma in the US" Zghebi SS, Mamas MA, Mohamed MO, Kontopantelis E. 2022

S3 Fig. Assessment of asthma admissions before and after the ICD code switch by calendar quarters 2012-2017

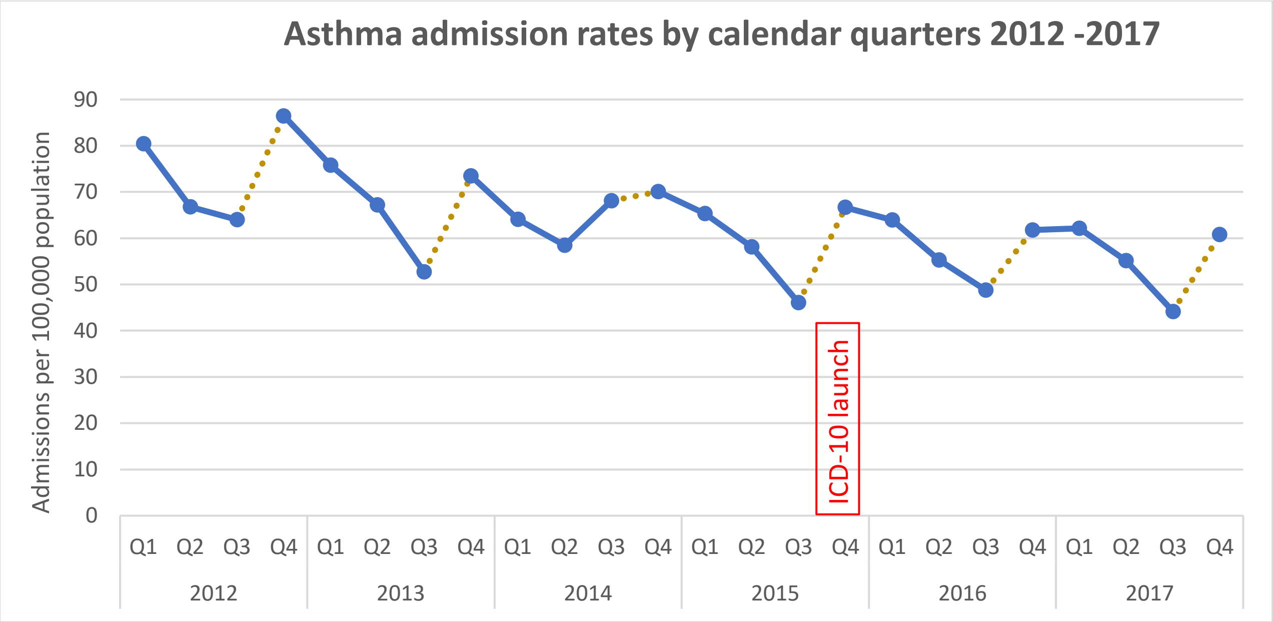

We observe an increase in admissions from 46 to 66.6/100,000 population following the code switch in October 2015. When further explored, we found that the **increase in admissions rate between Q3 and Q4** seems consistent in <u>years before and after</u> the code switch i.e. it appears to be unrelated to the code change from Q4 in 2015 (**dashed lines**). As reported in the paper, asthma admissions were overall higher during fall and winter (October-March), which is in line with previous literature attributing this to higher prevalence of respiratory infections and proneness to indoor air pollutants.
